# [Corrigendum] miR‑146b‑5p inhibits tumorigenesis and metastasis of gallbladder cancer by targeting Toll‑like receptor 4 via the nuclear factor‑κB pathway

**DOI:** 10.3892/or.2024.8699

**Published:** 2024-01-08

**Authors:** Bin Ouyang, Ningfeng Pan, Haifeng Zhang, Chuanming Xing, Wu Ji

Oncol Rep 45: 15, 2021; DOI: 10.3892/or.2021.7966

Following the publication of the article, the authors drew to the Editor's attention that, in Fig. 4D on p. 7, the data correctly shown to represent the E-cadherin bands for the “NOZ” experiment had inadvertently been used to show the Vimentin bands. However, the authors retained their original data, and the corrected version of Fig. 4, now showing the correct data for the Vimentin bands in Fig. 4D for the “NOZ” experiment, is shown on the next page. Note that this error did not grossly affect either the results or the conclusions reported in this work. All the authors agree with the publication of this Corrigendum, and are grateful to the Editor of *Oncology Reports* for granting them the opportunity to correct the error that was made during the assembly of this figure. Lastly, the authors apologize to the readership for any inconvenience this error may have caused.

## Figures and Tables

**Figure 4.**
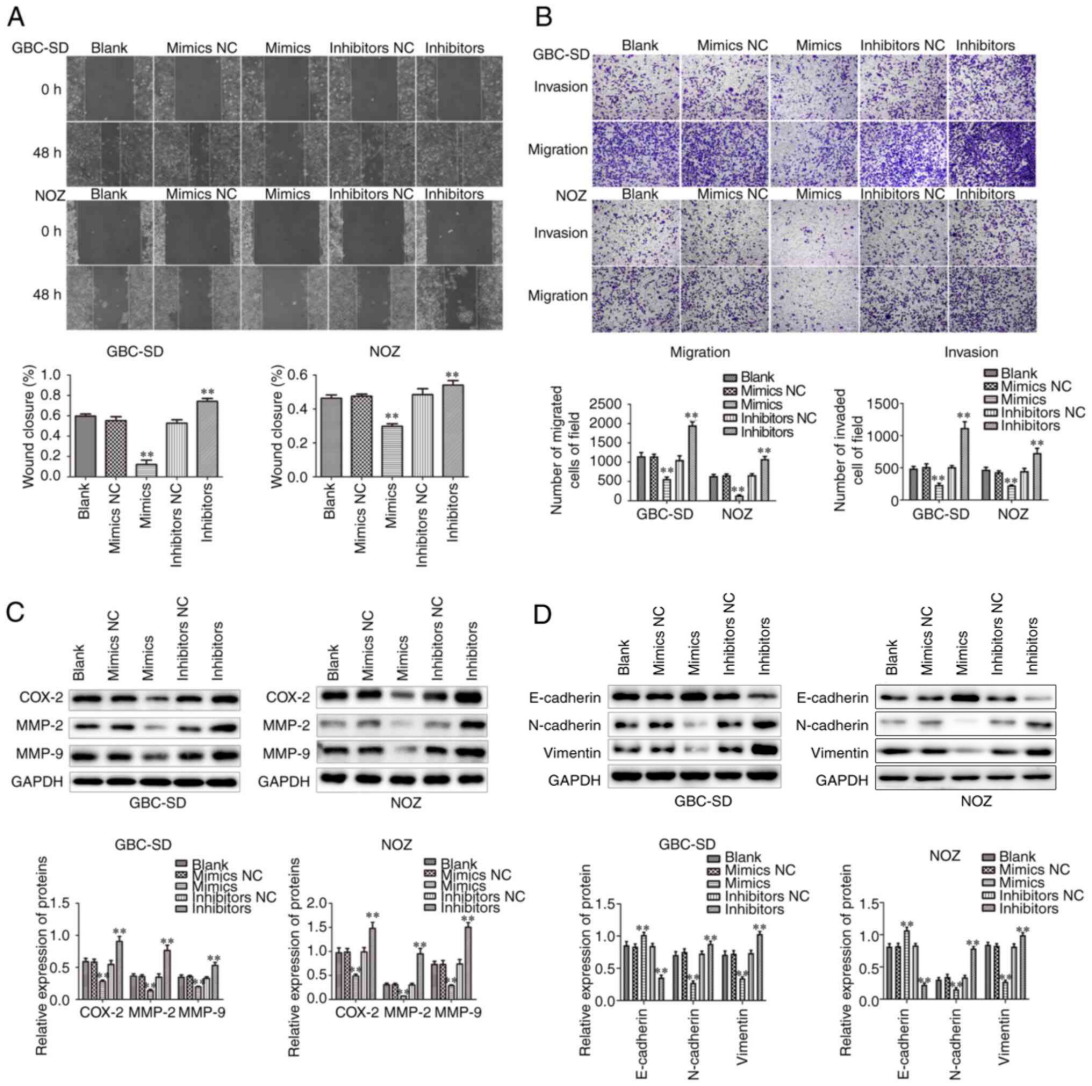
Effects of miR-146b-5p on the migration and invasion of GBC cells. (A and B) The migration and invasion of GBC-SD and NOZ cells in the miR-146b-5p inhibitor and miR-146b-5p mimic group were detected by wound healing assay and Transwell assay, respectively (magnification, ×200). (C) Western blotting was used to investigate the expression levels of the invasion-related proteins MMP-2, MMP-9 and COX-2 in GBC-SD and NOZ cells in different treatment groups. (D) The protein expression of E-cadherin, N-cadherin and vimentin expression was assessed by western blotting. All experimental data are expressed as mean ± SD and each assay was performed in triplicate. **P<0.01 vs. blank group. GBC, gallbladder cancer; MMP, matrix metallopeptidase; COX, cyclo-oxygenase.

